# A Case of Orofacial Granulomatosis Treated With Intravenous Rituximab

**DOI:** 10.7759/cureus.80123

**Published:** 2025-03-06

**Authors:** Sonia Selvaraj, Anuradha Priyadarshini, Krishnakanth Muralidhar, Leena Dennis Joseph, Adikrishnan Swaminathan

**Affiliations:** 1 Dermatology, Sri Ramachandra Institute of Higher Education and Research, Chennai, IND; 2 Pathology, Sri Ramachandra Institute of Higher Education and Research, Chennai, IND

**Keywords:** b cell role, biologicals, granulomatous cheilitis, orofacial granulomatosis, rituximab

## Abstract

Orofacial granulomatosis (OFG) encompasses a range of conditions marked by granuloma formation in the oral and perioral tissues. We report a 32-year-old female with asymptomatic swelling of the lower lip, buccal mucosa, and lower face, accompanied by difficulty in swallowing over a period of one year. A biopsy indicated OFG. The patient had an inadequate response to a combination of oral prednisolone, azathioprine, and hydroxychloroquine (HCQ). She was treated with three doses of injection rituximab resulting in a 50% reduction in swelling after six months. This case report aims to highlight the challenges of treating OFG, recommending rituximab as a promising therapeutic option, particularly when conventional treatments fail. Further research into biologics for this condition is warranted.

## Introduction

Orofacial granulomatosis (OFG) is a spectrum of conditions associated with granuloma formation in the oral and perioral tissues. OFG can be primary or secondary. Primary OFG includes granulomatous cheilitis and Melkersson-Rosenthal syndrome. Secondary OFG includes localized and systemic associations such as allergic contact dermatitis, foreign body reaction, sarcoidosis, and Crohn's disease [1]. In Miescher cheilitis, the granulomatous changes are confined to the lip, and this is generally regarded as a monosymptomatic form of Melkersson-Rosenthal syndrome. Melkersson-Rosenthal syndrome comprises recurrent orofacial swelling, facial palsy, and plicated tongue. Dietary antigens such as cinnamic aldehyde, benzoates, butylated hydroxyanisole, dodecyl gallate, and menthol, and conditions such as atopy, Crohn's disease, sarcoidosis, cobalt reaction, and paratubercular and mycobacterial stress protein can predispose individuals to OFG [2]. This case report discusses rituximab's successful treatment of OFG when conventional treatments fail.

## Case presentation

**Figure 1:**
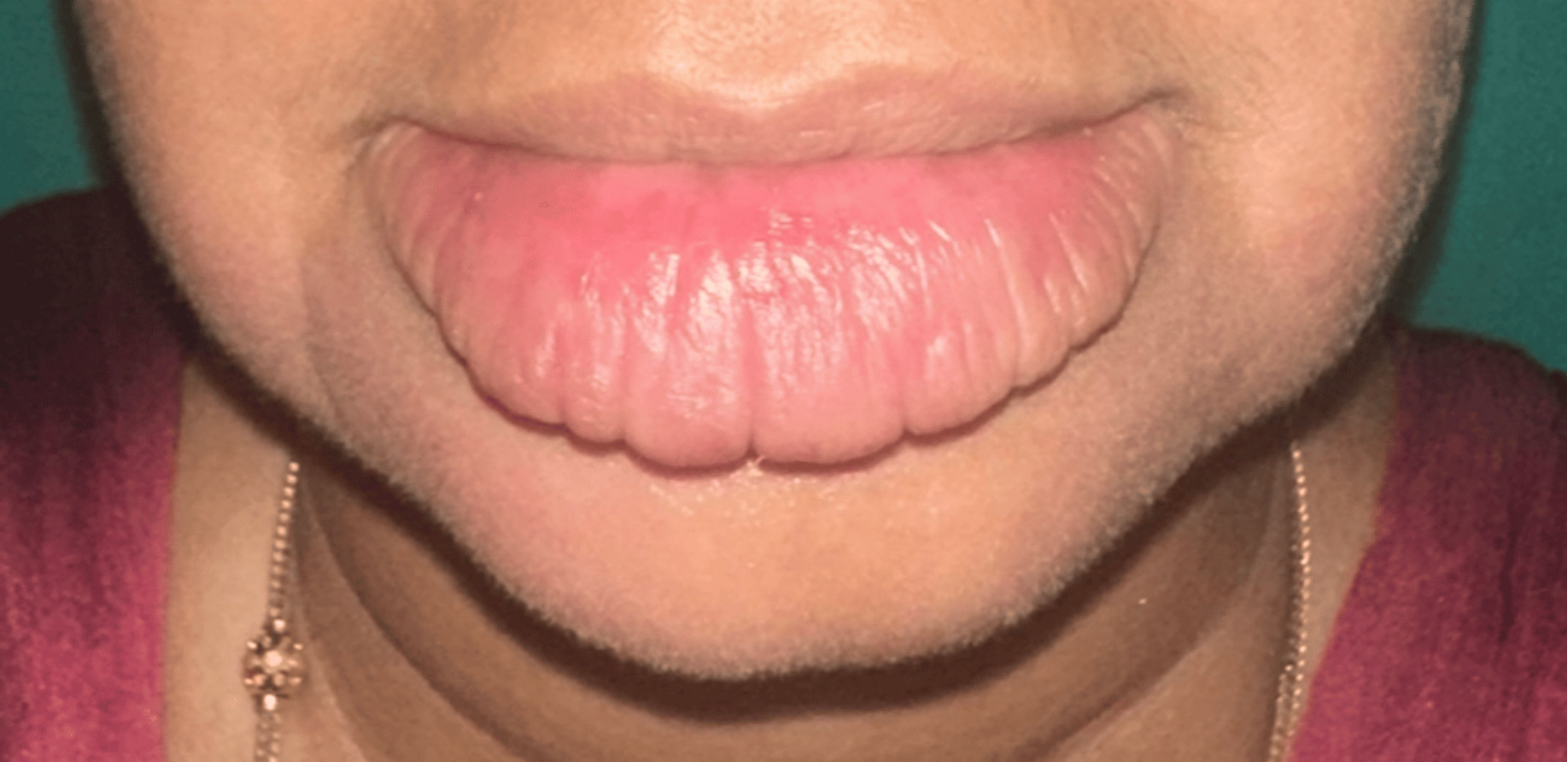
Diffuse firm indurated swelling of the lower lip and skin of the chin

**Figure 2:**
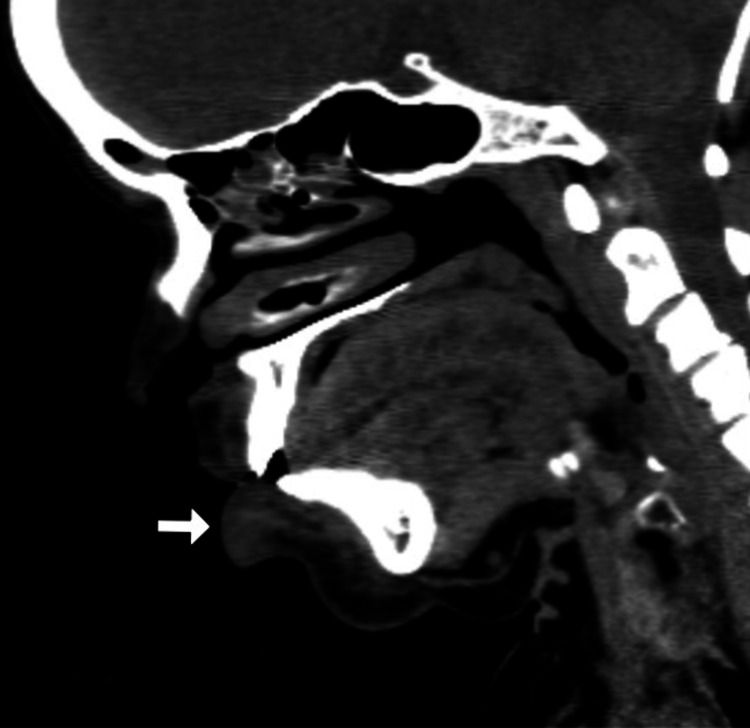
CT of the head and neck (sagittal view) showing diffuse soft tissue swelling in the lower lip (white arrow) CT: computed tomography

**Figure 3:**
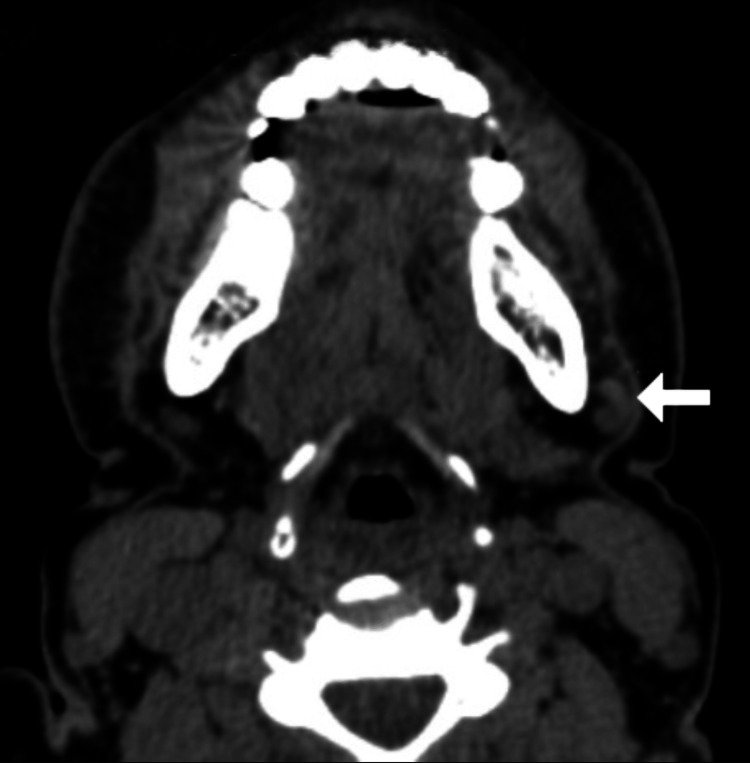
CT of the head and neck (axial view) showing multiple cervical lymph nodes (white arrow) CT: computed tomography

**Figure 4:**
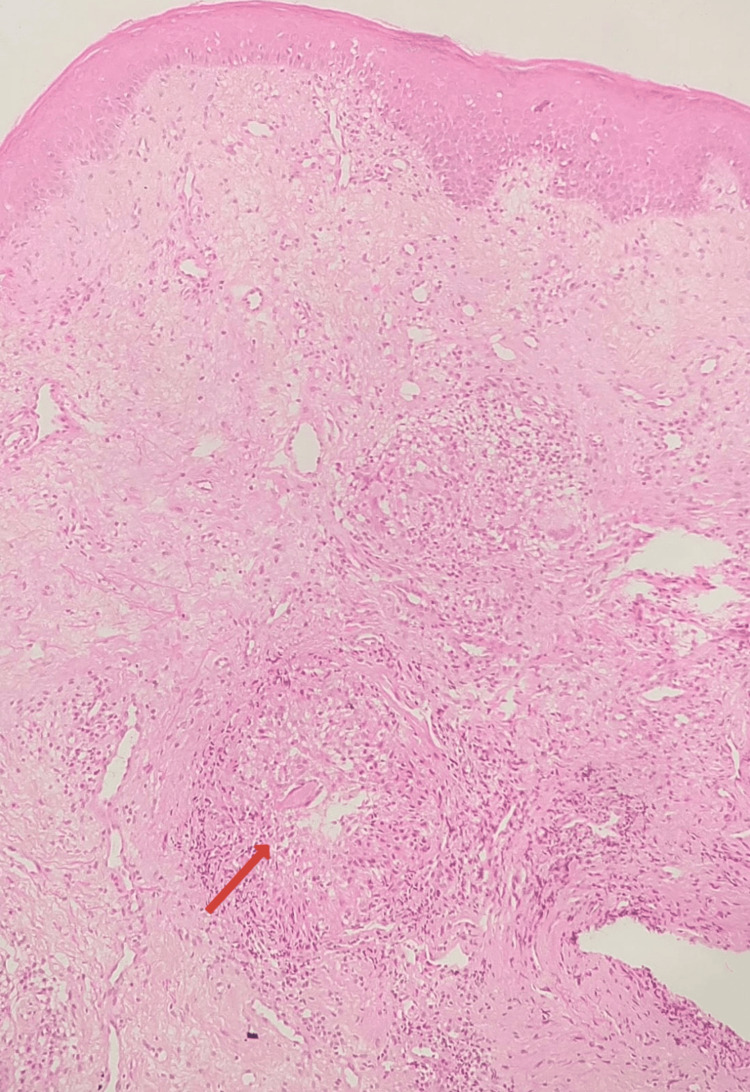
Histopathology image of the biopsy from the lower lip Stratified squamous epithelium with epithelioid granulomas (red arrow) in the deep dermis (hematoxylin and eosin stain, 100×)

**Figure 5:**
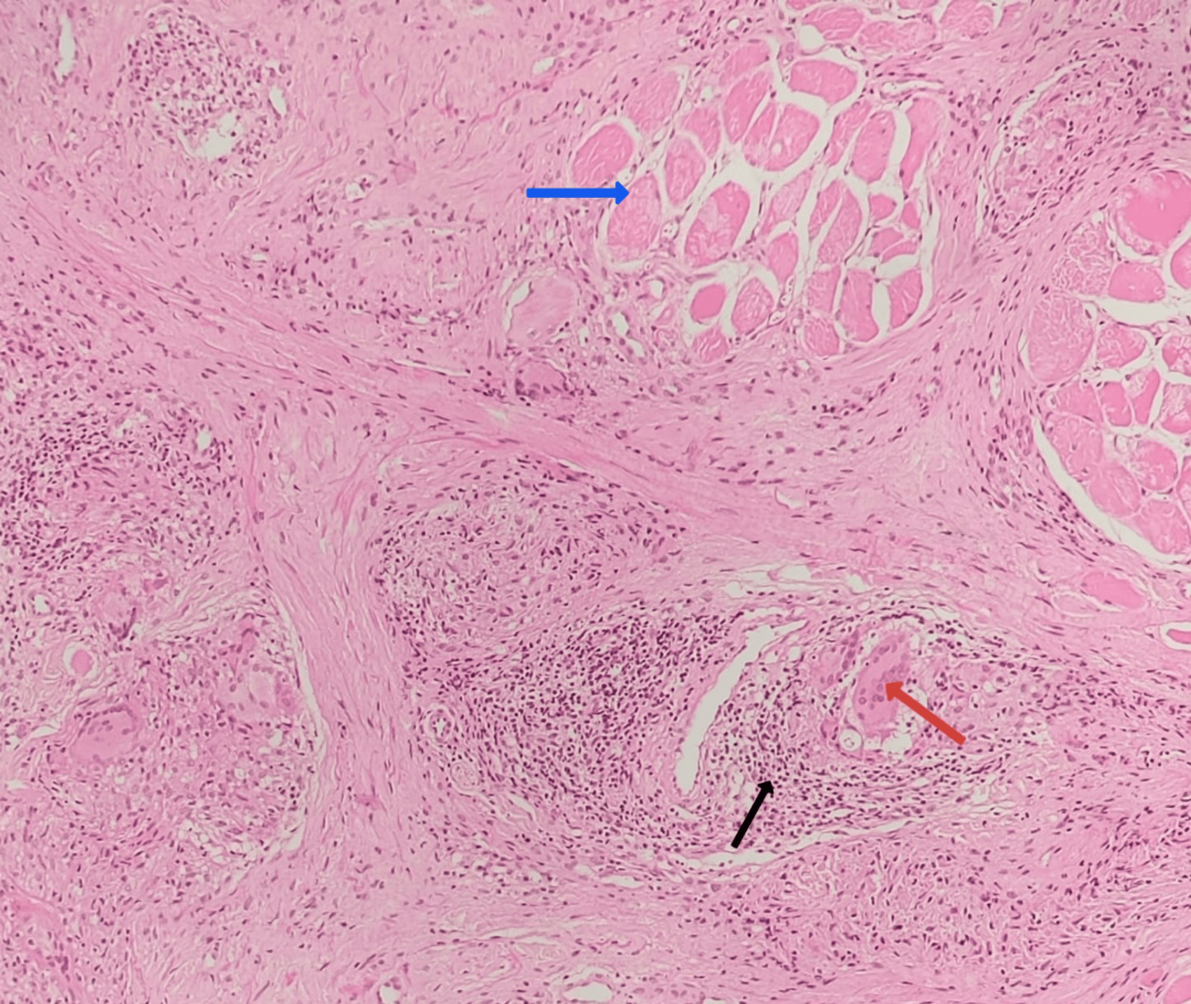
Histopathology image of the biopsy from the lower lip Epithelioid granulomas with foreign body giant cells (red arrow) and lymphocytes (black arrow) surrounding the skeletal muscle bundles (blue arrow) (hematoxylin and eosin stain, 200×)

A 32-year-old female patient presented with persistent, progressive, asymptomatic indurated swelling of the orofacial region involving the buccal mucosa, lower lip, and chin of one-year duration (Figure 1). The differential diagnoses considered were Crohn's disease, sarcoidosis, and tuberculosis. Laboratory investigations revealed an elevated erythrocyte sedimentation rate; normal levels of serum calcium, vitamin D, and angiotensin-converting enzyme (ACE) levels; negative Mantoux test; and negative fecal calprotectin (Table 1). A computed tomography (CT) scan of the head and neck showed diffuse soft tissue swelling of the lower lip along with cervical lymphadenopathy (Figures 2, 3). The chest radiograph was normal. A biopsy taken from the lip revealed normal epidermis, epithelioid granulomas, foreign body giant cells, and numerous lymphocytes in the dermis and around the underlying muscle fiber bundles. No necrosis was noted. Multiple sections of the biopsy were examined for fungal elements, but none were detected (Figures 4, 5). Ziehl-Neelsen (ZN) stain of histopathological examination (HPE) was negative.

**Table 1:** Investigation values of the patient

Investigations	Values	Normal/range values
Erythrocyte sedimentation rate	19 mm/hour	4-12 mm/hour
Serum calcium	9.2 mg/mL	8.8-10.6 mg/dL
Serum vitamin D	32.76 ng/dL	30-70 ng/dL
Serum angiotensin-converting enzyme	49 U/L	13.3-63.9 U/L
Fecal calprotectin (quantitative)	Negative	<50 ug/g: negative, 50-200 ug/g: borderline, >200 ug/g: positive

Normal serum calcium, ACE levels, and absence of naked granulomas in HPE excluded sarcoidosis. A negative Mantoux test, normal chest radiograph, and negative Ziehl-Neelsen stain of HPE ruled out tuberculosis. The absence of fungal elements in multiple sections of the biopsy ruled out deep fungal infections. Negative fecal calprotectin test and absence of gastrointestinal symptoms excluded Crohn's disease. Based on histopathological findings of granulomatous cheilitis and ruling out systemic associations, a final diagnosis of orofacial granulomatosis (OFG) was made.

**Figure 6:**
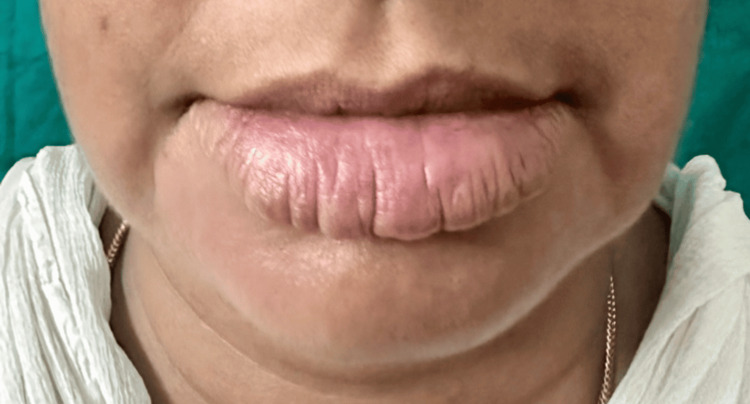
Post-treatment clinical photograph showing a 50% reduction in the size of the lip swelling after two doses of injection rituximab

The patient was initially treated with a daily regimen of 20 mg oral prednisolone, 200 mg hydroxychloroquine (HCQ), and 100 mg azathioprine. However, after four weeks of treatment, there was no significant reduction in the size of the swelling, indicating an inadequate clinical response. Given the refractory nature of the condition, the patient was considered a candidate for rituximab therapy. She received two doses of intravenous rituximab of 500 mg each administered 14 days apart. Following this treatment, there was a 50% reduction in the size of the swelling, as demonstrated in Figure 6. A third dose of intravenous 500 mg rituximab was administered six months later. Subsequently, the steroid therapy was gradually tapered and eventually discontinued. At present, the patient is being maintained on azathioprine and hydroxychloroquine, with continued clinical follow-up.

## Discussion

OFG encompasses a range of conditions marked by granuloma formation in the oral and perioral tissues [1]. The true prevalence of the condition is 0.8%, affecting children and young adults, common in both genders with slight female predilection [3,4]. The symptoms may include perioral erythema, intermittent swelling of one or both lips, and persistent swelling with a stiff, rubbery texture of the lips. Eventually, the lips develop angular cheilitis and persistent edema linked to infection, such as *Staphylococcus aureus* or *Candida*. Mucosal manifestations include gingivitis, linear ulcers, mucosal tags, and a cobblestone appearance [2].

First-line treatment options are topical/intralesional steroids. Other treatment options include systemic corticosteroids, oral antibiotics (e.g., minocycline, metronidazole, and clofazimine), adalimumab, methotrexate, and dapsone. Refractory cases have been managed surgically, with reduction cheiloplasty [1].

The majority of systemic medications were at least somewhat successful in OFG, according to a case series of OFG by Jaouen et al. [5], although only 20% of cases had a complete response or an improvement of more than 70%, with a brief duration of remission [6].

The role of B cells in granuloma formation is pivotal, particularly in chronic inflammatory conditions such as OFG. B cells contribute to this process through multiple mechanisms, including antibody production, cytokine secretion, antigen presentation, and memory response. Antibody production involves the transformation of B lymphocytes into plasma cells, which generate antibodies that neutralize infectious agents, mediate antibody-dependent cellular cytotoxicity (ADCC), activate the complement system, and enhance bacterial phagocytosis and apoptosis [7]. Additionally, B cells secrete a diverse range of cytokines, from pro-inflammatory cytokines such as interleukin-6 (IL-6) to immunosuppressive cytokines such as interleukin-10 (IL-10), thereby supporting granuloma formation [8]. Through antigen presentation, B cells facilitate antigen-specific T-cell activation by cross-linking antigen receptors and attracting T-cell help, thereby intensifying the immune response [9]. Moreover, the generation of memory B cells ensures a rapid and robust response to previously encountered pathogens, which significantly influences the persistence of granulomas in chronic infections [10,11].

Rituximab, a chimeric anti-CD20 monoclonal antibody (MAb), induces the killing of normal and malignant B cells expressing the cell-surface molecule CD20 [12]. Thus, using rituximab will lead to a reduction of autoantibodies and alter the cytokine environment affecting the balance between pro-inflammatory and anti-inflammatory signals in the formation of granuloma.

In the case series of OFG by Jaouen et al. [5], treatment with rituximab showed a complete response of 70% or more swelling reduction or a complete return to normal size [6]. A case report on Wegener's granulomatosis with an intraoral presentation by Staines et al. was successfully treated with rituximab [13].

## Conclusions

Orofacial granulomatosis (OFG) can be refractory to treatment with conventional immunomodulators, posing a significant challenge in management. Rituximab, a monoclonal antibody targeting B cells, offers a promising therapeutic option by modulating immune responses. When combined with other immunomodulators, it targets multiple pathogenic pathways, leading to improved clinical outcomes. Its use has been associated with significant symptom relief and a reduction in swelling, particularly in treatment-resistant cases. Additionally, rituximab helps minimize the need for prolonged corticosteroid use, thereby reducing associated side effects. Continued research will help refine individualized treatment approaches, ultimately enhancing patient outcomes and quality of life.
